# Primary lymphoepithelial carcinoma of the intraoral minor salivary gland: A case report

**DOI:** 10.3892/ol.2014.2755

**Published:** 2014-12-02

**Authors:** MING ZENG, SHUANGJIANG LI, JINHUA FU, HANJIANG WU, YIJUN GAO

**Affiliations:** Department of Stomatology, Second Xiangya Hospital, Central South University, Changsha, Hunan 410011, P.R. China

**Keywords:** lymphoepithelial carcinoma, minor salivary gland, Epstein-Barr virus

## Abstract

Lymphoepithelial carcinoma (LEC) of salivary gland origin is a rare malignant tumor with morphological characteristics identical to those of undifferentiated nasopharyngeal carcinoma. It has a marked racial predilection for Inuit and Southeast Asian populations. An association between LEC and Epstein-Barr virus (EBV) infection has previously been reported. LEC most frequently affects the parotid gland, followed by the submandibular glands. To the best of our knowledge, only three LECs arising from the minor salivary gland have been previously described in the English language literature. The current study reports a case of EBV-associated LEC of the minor salivary gland in the hard palate of a 38-year-old Chinese female, and reviews the clinicopathological characteristics of this uncommon tumor.

## Introduction

Lymphoepithelial carcinoma (LEC) of salivary gland origin is an undifferentiated carcinoma accompanied by a prominent non-neoplastic lymphoplasmacytic infiltrate; LEC exhibits morphological characteristics identical to those of nasopharyngeal carcinoma (1). It has a significant racial predilection for Inuit and Southeast Asian populations (1,2). An association between LEC and Epstein-Barr virus (EBV) infection has been previously reported (1,2). The majority of cases of LEC arise in major salivary glands, with the parotid being most commonly affected (~80% of cases) (2). LEC presents as slowly growing mass which may exhibit a rapid increase in size with or without pain. Cervical lymph node involvement is present in ~40% of patients at presentation (2,3). Due to the lack of distinctive clinical and radiographical features and the rarity of the tumor, the diagnosis of LEC is initially based on histology. Clinical evaluation for possible primary nasopharyngeal carcinoma must be performed in all patients. At present, treatment for LEC includes complete surgical excision and adjuvant radiotherapy. To the best of our knowledge, only three cases of LEC have been reported in the English language literature as arising from the minor salivary gland (4–6). The current study presents the fourth such case of LEC originating from the minor salivary gland of the hard palate, and reviews the clinicopathological characteristics of this uncommon tumor.

## Case report

This study was approved by the Ethics Committee of The Second Xiangya Hospital, Central South University (Hunan, China) and written informed consent was obtained from the patient.

**Figure 1:**
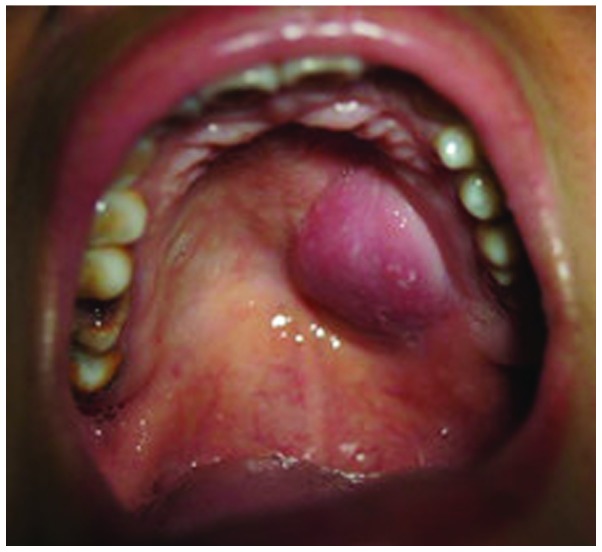
Intraoral image showing a nodular submucosal tumor on the left side of the hard palate.

**Figure 2:**
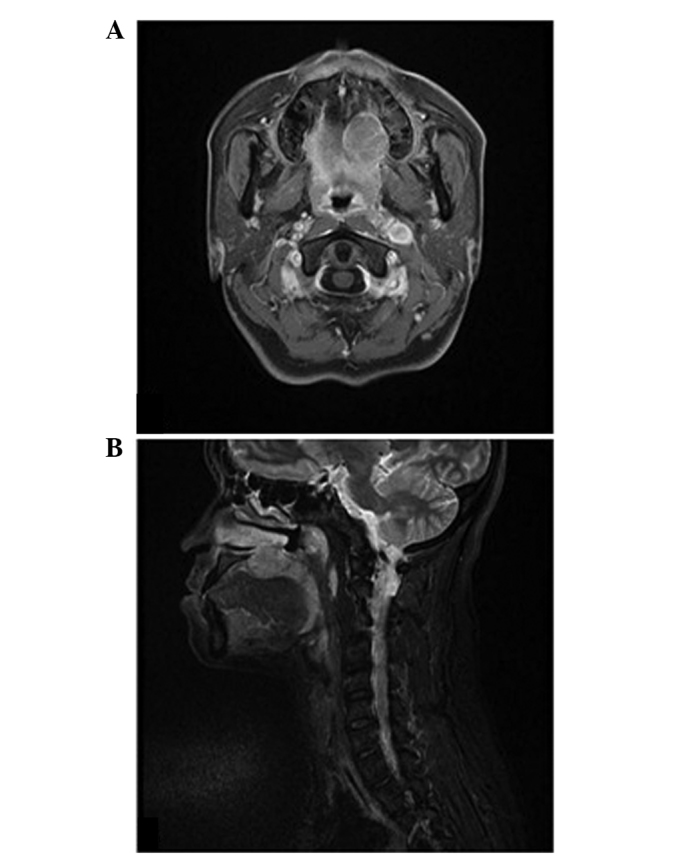
(A) Axial and (B) sagittal magnetic resonance images show a mass of 26 × 24 × 17 mm in size, located on the left hard palate with invasion into the nasal cavity.

**Figure 3:**
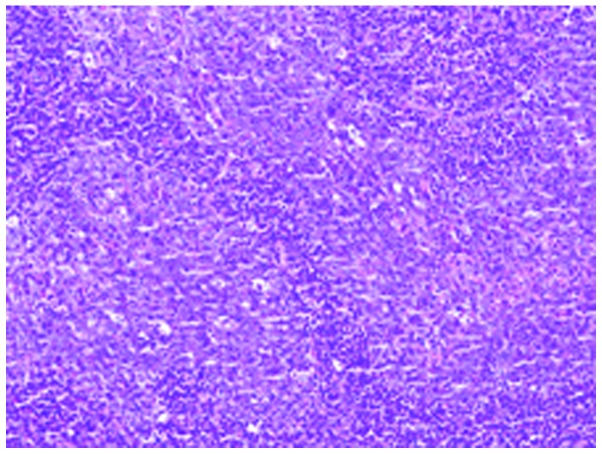
Histopathology of the lymphoepithelial carcinoma showing tumor cells with vesicular nuclei and prominent nucleoli intermingled with lymphocytes and plasma cells (staining, hematoxylin and eosin; magnification, ×200).

**Figure 4:**
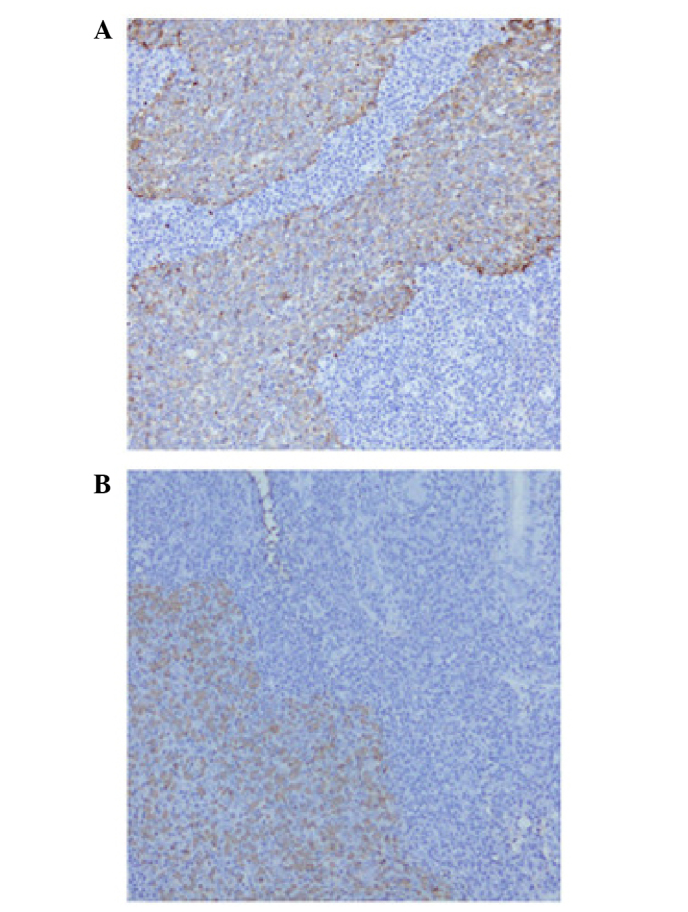
(A) Tumor cells tested positive for cytokeratin AE1/AE3 (immunohistochemical stain; magnification, ×200). (B) Tumor cell nuclei tested positive for Epstein-Barr virus encoded RNAs. Note: The surrounding lymphocytes and adjacent salivary gland tissues are negative (EBER *in situ* hybridization; magnification, ×200).

A 38-year-old Chinese female was admitted to the Department of Stomatology at the Second Xiangya Hospital, of Central South University (Changsha, China) with a painless mass in her left palate. The patient stated that the mass was initially identified approximately one month previously. Intraoral examination revealed a well-defined, nodular mass in the left side of the hard palate (Fig. 1). The mass was non-tender and soft to palpation, with areas of fluctuance. The surface mucosa was red in color with no erosion, bleeding or ulceration. Magnetic resonance imaging delineated a mass lesion of 26 × 24 × 17 mm in size, located on the left hard palate with invasion into the nasal cavity (Fig. 2). The tumor exhibited isointensity on T1-weighted images and hyperintensity on T2-weighted images, with a moderately enhanced effect. No marked lymphadenopathy was identified according to imaging features and clinical examination. The patient was otherwise well, with no significant events in the medical history. An incisional biopsy was performed, which revealed irregular tumor nests of undifferentiated epithelial cells intimately intermingled with lymphocytes and plasma cells. The tumor cells exhibited a syncytial pattern with indistinct cell borders, vesicular nuclei, and large central nucleoli (Fig. 3). Immunohistochemically, the tumor cells were diffusely positive for cytokeratin AE1/AE3 (Fig. 4A). *In situ* hybridization for EBV-encoded RNA was diffusely positive in undifferentiated carcinoma, however, it was negative in the surrounding lymphoid stroma and adjacent salivary gland tissues (Fig. 4B). Endoscopy examination revealed a thickening of the nasopharynx. Multiple biopsies from the nasopharynx were subsequently performed, which were negative for tumor cells. A final diagnosis of primary LEC was determined. The patient underwent a partial maxillectomy without further surgical neck dissection. Adjuvant radiotherapy was suggested, however this was refused by the patient due to financial difficulties. The patient showed no evidence of remission during the postoperative follow-up of 12 months.

## Discussion

According to the new World Health Organization classification, undifferentiated carcinomas of the salivary gland are separately classified into small-cell undifferentiated carcinoma, large-cell undifferentiated carcinoma and LEC (1). LEC, is a rare and unique malignant salivary gland tumor with morphological features identical to that of undifferentiated nasopharyngeal carcinoma, but which are not shared by other salivary gland tumor types (1,2). This neoplasm exhibits a strong racial prevalence for Inuit and Southeast Asian individuals, however the reasoning for this remains unknown. Within the Inuit population, LEC is the most common malignant salivary gland tumor, representing 92% of all cases (7). LEC accounts for 5.9 and 5.4% of malignant salivary gland tumors in Southeast Chinese and Taiwanese populations, respectively (3,8). In nonendemic areas, LEC accounts for ~0.4% of malignant tumors in the salivary gland (2). An association between LEC and Epstein-Barr virus (EBV) infection has also been previously reported (1,2). In endemic areas, the association between LEC occurring in the salivary glands and EBV infection is ~100% (3,6). The age range at presentation is between 20 and 60 years, with a median age of 40 years. There is no evident gender predilection (3).

Although LEC may occur in any salivary gland location, the diagnosis of a primary LEC arising in a salivary gland is largely restricted to the major glands due to difficulties with differentiating LEC from tumors of primary mucosal origin. The parotid gland is most frequently affected, followed by the submandibular gland (2). Primary LEC of minor salivary gland origin is extremely rare, being absent in recent large cohort of salivary gland LECs and several previous studies of minor salivary gland tumors (3,8–10). It is conceivable, however, that the incidence of LEC of the minor salivary glands may in fact be higher; this is due to difficulties in reliably distinguishing them from tumors of primary mucosal origin, therefore, by convention they are not included in the majority of current salivary gland neoplasm classifications. In the present case, the distance from the overlying nondysplastic epithelium and close proximity to glandular tissue strongly indicated a primary minor salivary gland origin. To the best of our knowledge, only four cases of intraoral LEC of presumed minor salivary gland origin, including the present case, have been reported (4–6). The four cases involved three females and one male; the mean age was 53 years and the age range was 38–69 years. Two cases arose in the cheek, and one each in the palate and upper lip. The tumors varied in diameter from 0.5–2.6 cm, with a mean diameter of 1.9 cm. Lymph node metastasis was observed in one patient. Three patients were treated with surgery, and one was treated with surgery followed by adjuvant radiotherapy. Patient follow-up varied from 12–120 months. All patients were alive with no evidence of disease or recurrence during follow-up. EBV infection was identified in two patients who were from Southeast Asia.

LEC is a lymphoid rich tumor composed of sheets and nests of large vesicular cells with prominent nucleoli and syncytial cytoplasm. The principle differential diagnosis of the present case is metastatic undifferentiated carcinoma, particularly metastasis from the nasopharynx. Morphologically, LEC of the salivary gland is indistinguishable from nasopharyngeal carcinoma, which is much more common, and has very similar cytological and architectural features, the same ethnic predilection, and a strong association with EBV infections. Therefore, a careful examination of the nasopharynx is mandatory and, if relevant, multiple nasopharyngeal biopsies must be obtained (1,2).

LEC has a tendency to metastasize, and the reported incidence of lymph node metastases of LECs originating from the major salivary glands at presentation is 10–50% (1–3,8). In 20% of cases, local recurrences or lymph node metastases develop, and ~20% eventually experience distant metastases within three years of treatment. Appropriate therapy for LEC includes complete surgical excision with negative surgical margins and adjuvant radiation therapy to the tumor area and ipsilateral neck nodes. A neck dissection is usually reserved for patients with clinically positive lymph nodes. LECs appear to exhibit an improved prognosis compared with other undifferentiated carcinomas of the salivary glands, and the 5-year survival has been reported to range from 50–90% (1–3,8).

Due to the limited number of cases, no standardized treatment policy for LEC of the oral cavity has been established to date. We hypothesize that the initial treatment choice for oral LEC should be complete local excision, accompanied by neck lymphadenectomy (if cervical nodes are involved). Adjuvant radiotherapy must be considered for LEC due to its high sensitivity, particularly in patients with extensive soft tissue invasion, in cases in which tumor-free margins are not possible, or in patients with local recurrence.
